# Xenoestrogen-Induced ERK-1 and ERK-2 Activation via Multiple Membrane-Initiated Signaling Pathways

**DOI:** 10.1289/ehp.7175

**Published:** 2004-07-28

**Authors:** Nataliya N. Bulayeva, Cheryl S. Watson

**Affiliations:** Department of Human Biological Chemistry and Genetics, University of Texas Medical Branch, Galveston, Texas, USA

**Keywords:** environmental estrogens, ERKs, estradiol, phytoestrogens, prolactinoma cell line, rapid estrogen effects

## Abstract

Xenoestrogens can mimic or antagonize the activity of physiological estrogens, and the suggested mechanism of xenoestrogen action involves binding to estrogen receptors (ERs). However, the failure of various *in vitro* or *in vivo* assays to show strong genomic activity of xenoestrogens compared with estradiol (E_2_) makes it difficult to explain their ability to cause abnormalities in animal (and perhaps human) reproductive functions via this pathway of steroid action. E_2_ has also been shown to initiate rapid intracellular signaling, such as changes in levels of intracellular calcium, cAMP, and nitric oxide, and activations of a variety of kinases, via action at the membrane. In this study, we demonstrate that several xenoestrogens can rapidly activate extracellular-regulated kinases (ERKs) in the pituitary tumor cell line GH_3_/B6/F10, which expresses high levels of the membrane receptor for ER-α(mER). We tested a phytoestrogen (coumestrol), organochlorine pesticides or their metabolites (endosulfan, dieldrin, and DDE), and detergent by-products of plastics manufacturing (*p*-nonylphenol and bisphenol A). These xenoestrogens (except bisphenol A) produced rapid (3–30 min after application), concentration (10^−14^–10^−8^ M)-dependent ERK-1/2 phosphorylation but with distinctly different activation patterns. To identify signaling pathways involved in ERK activation, we used specific inhibitors of ERs, epidermal growth factor receptors, Ca^2+^ signaling, Src and phosphoinositide-3 kinases, and a membrane structure disruption agent. Multiple inhibitors blocked ERK activation, suggesting simultaneous use of multiple pathways and complex signaling web interactions. However, inhibitors differentially affected each xenoestrogen response examined. These actions may help to explain the distinct abilities of xenoestrogens to disrupt reproductive functions at low concentrations.

Xenoestrogens are a large and structurally diverse group of compounds, which can act as inappropriate estrogens and/or can interfere with the actions of endogenous estrogens such as estradiol (E_2_) or other reproductive steroids. Many studies have demonstrated that contamination of the environment or treatment with xenoestrogens can disrupt developmental programs to alter sexual phenotypes and reproductive functions. Examples of such disruptions are a decline in the sperm quality of fish, interference with the sexual development of alligators and turtles, disruption of pregnancies in laboratory animals, interference with blastocyst implantation, and inappropriately induced progesterone receptor expression and uterine weight increases [reviewed in Witorsch (2002)]. In addition, environmental estrogens have been shown to inhibit the human sperm acrosomal reaction (Turner et al. 1997), and xenoestrogens are also suspected of causing breast cancer cell and vaginal epithelial cell proliferation (Krishnan et al. 1993; Long et al. 2000).

Most previous studies investigated the possible actions of xenoestrogens through classical nuclear estrogen receptors (ERs) modifying gene activity (Long et al. 2000; Massaad and Barouki 1999; McLachlan 1993; Rosselli et al. 2000; Sonnenschein et al. 1995). A variety of *in vivo* and *in vitro* screening assays have been applied to predict the estrogenic potential of xenoestrogens, including several receptor-reporter-gene assay systems in transfected mammalian or yeast cell lines (Bonefeld-Jorgensen et al. 2001; Elsby et al. 2001; Gutendorf and Westendorf 2001; Hodges et al. 2000; Inoue et al. 2002; Lee et al. 2002; Massaad and Barouki 1999; Rajapakse et al. 2002; Willard and Frawley 1998). However, these methods demonstrated that xenoestrogens act very weakly (only at concentrations 1,000- to 10,000-fold higher than E_2_), if at all, via genomic activation pathways. Other tests have been assumed to assess genomic responses, but may in fact be assays for either nongenomic or combination mechanisms. These include cell proliferation test systems (Gutendorf and Westendorf 2001; Hodges et al. 2000; Rousseau et al. 2002; Soto et al. 1994), receptor binding assays for ER-α or ER-β (Granek and Rishpon 2002; Gutendorf and Westendorf 2001; Lee et al. 2002), and predictions of ligand binding affinity and conformation of ER complexes with xenoestrogens by computer modeling (Suzuki et al. 2001; van Lipzig et al. 2004; Yu et al. 2002).

In addition to the classical genomic pathway, steroids can produce rapid (within a few minutes after application) nongenomic signaling effects via second messenger systems, for example, Ca^2+^, K^+^, cAMP, and nitric oxide level changes; activation of G protein–mediated events; and stimulation of different types of kinases such as extracellular-regulated kinases (ERKs), phosphoinositide-3 kinase (PI3K), p38, and Jun kinase (Junk) (Aronica et al. 1994; Doolan and Harvey 2003; English et al. 1999; Filardo et al. 2000; Haynes et al. 2003; Kelly et al. 1999; Nadal et al. 1998; Prevot et al. 1999; Razandi et al. 2003). Although the precise molecular mechanisms of nongenomic actions are not fully understood, it is known that some rapid E_2_ effects can be initiated by ligand binding to membrane-associated ERs (mERs) that have been shown to be the same proteins as their nuclear receptor counterparts in several systems (Chen et al. 1999; Levin 1999; Norfleet et al. 1999; Pappas et al. 1994). Therefore, it is reasonable to suggest that xenoestrogens can bind to mER and produce rapid changes in signaling, similar to E_2_. However, there are few data (Adeoya-Osiguwa et al. 2003; Nadal et al. 2000; Ruehlmann et al. 1998; Sato et al. 2003; Watson et al. 1999a; Wober et al. 2002) addressing the ability of environmental estrogens to mediate nongenomic estrogenic actions, and many studies on this phenomenon have used concentrations of xenoestrogens much higher than would be found in contamination sites.

In the present study, we investigated the ability of some of these estrogen mimetics (belonging to the major classes of environmental estrogens) to produce rapid activation of ERKs via various signaling pathways in the GH_3_/B6/F10 prolactinoma cell line. We previously selected this subline for its robust expression of mER-α and its capacity for rapid E_2_-induced prolactin release (Pappas et al. 1994), and we also demonstrated that adequate levels of mER were necessary to elicit rapid ERK responses (Bulayeva et al. 2004). Here, we tested organochlorine pesticides [dieldrin, endosulfan, and the DDT metabolite *o*,*p*′-dichlorodiphenylethylene (DDE)], detergents used in plastics manufacturing (*p*-nonylphenol and bisphenol A), and the natural phytoestrogen coumestrol. The signaling pathways that we examined are generally known to initiate at the plasma membrane and go through multiple steps before converging on the ERKs. Others have associated features such as G protein involvement, Ca^2+^ influx, and epidermal growth factor receptor (EGFR) phosphorylation with downstream ERK activations, which can lead to diverse cellular functions such as cell proliferation, transformation, differentiation, and migration (Belcheva and Coscia 2002; Razandi et al. 2003). Xenoestrogens, such as endosulfan and chlordane (Cossette et al. 2002) and nonylphenol, bisphenol A, and coumestol (Gutendorf and Westendorf 2001), at relatively low (10^−9^–10^−7^ M) concentrations, can produce proliferation in other cell lines, and this function can be regulated by the xenoestrogen benzopyrene through ERK (Tsai et al. 2004), The alteration of cell proliferation, as well as other functions downstream of ERK activation, could lead to endocrine disruptions known to be caused by environmental estrogens.

To monitor signaling events leading to ERK activation, we used our fixed cell–based ELISA (Bulayeva et al. 2004), which allows us to analyze many samples and thus detailed time- and concentration-dependent changes in ERK phosphorylation resulting from actions of multiple xenoestrogenic compounds and specific inhibitors of signaling cascade participants. Such comparative explorations of differential signaling pathway involvement, kinetics, and potencies unique to each compound may lead to more detailed predictions about the hazards of exposure mediated through different subsets of responses related to endocrine disruption by individual xenoestrogens.

## Materials and Methods

Phenol red–free Dulbecco’s modified Eagle medium (DMEM) was purchased from Mediatech (Herndon, VA). Horse serum was obtained from Gibco BRL (Grand Island, NY); defined supplemented calf sera and fetal bovine sera were from Hyclone (Logan, UT). Endosulfan and DDE were purchased from Ultra Scientific (North Kingstown, RI). From Vector Laboratories (Burlingame, CA), we purchased biotinylated universal anti-mouse/rabbit IgG, Vectastain ABC-AP (avidin:biotinylated enzyme complex with alkaline phosphatase) detection systems, levamisol (endogenous alkaline phosphatase subtype inhibitor), and *para*-nitrophenol phosphate (pNpp; the substrate for our alkaline phosphatase reaction). Phospho-p44/42 ERK (pERK) monoclonal antibody (Ab), anti-mouse horseradish peroxidase–linked Ab, and lysis buffer were obtained from Cell Signaling Technology (Beverly, MA). Paraformaldehyde was from Fisher Scientific (Fair Lawn, NJ). BAPTA-AM (B-TA) was from Molecular Probes (Eugene, OR), and PP2, Ag 1468 (AG 14), and Ly294002 (Ly) were from Calbiochem (San Diego, CA). ICI 182,780 (ICI) was from Tocris (Ellisville, MO). All other reagents were purchased from Sigma Chemical Company (St. Louis, MO).

### Cell culture.

Our clonal rat prolactinoma cell line GH_3_/B6/F10 was selected for high expression of mER-α (Pappas et al. 1994). Cells were routinely subcultured in DMEM containing 12.5% horse serum, 2.5% defined supplemented calf serum, and 1.5% fetal calf serum. For individual experiments, cells were deprived of steroids for 48 hr after plating by substituting DMEM containing 1% charcoal-stripped (4×) serum. All test estrogens were dissolved in ehanol (EtOH) at a 10^−2^ M concentration to create a stock solution and then diluted into experimental media to yield final concentrations from 10^−8^ to 10^−12^ M. The EtOH concentration used as the vehicle control was 0.0001%.

### Fixed cell–based ELISA.

To estimate ERK phosphorylation quantitatively, we used a cell-based ELISA, which we previously developed and described (Bulayeva et al. 2004). Briefly, cells (10^4^ cells/well) were plated in 96-well plates (Corning Incorporated, Corning, NY) and withdrawn from serum hormones by incubation in medium containing 1% charcoal-stripped serum for 48 hr before experiments began. The cells were next treated with hormones and estrogen mimetics for 3–30 min, and then fixed with 2% paraformaldehyde/0.2% picric acid at 4°C for 48 hr. After fixation, the cells were incubated with phosphate-buffered saline (PBS) containing 2% bovine serum albumin (BSA) and 0.1% Triton X-100 for 1 hr at room temperature (RT), and then with primary Ab against pERK (1:400 in PBS/1% BSA/0.1% Triton X-100) overnight at 4°C. After a wash with PBS, biotin-conjugated secondary Ab (1:300) in PBS/1% BSA was added for 1 hr at RT. The cells were again washed in PBS and incubated with Vectastain ABC-AP solution (100 μL/well) for 1 hr at RT, and then Vectastain alkaline phosphatase substrate (pNpp solution) with levamisole was added to each well (100 μL). Plates were incubated in the dark for 30 min at 37°C, and the signal from *para*-nitrophenol (pNp) was read at A_405_ in a model 1420 Wallac microplate reader (Perkin Elmer, Boston, MA). The pNp signal was normalized to cell number, determined by using the crystal violet (CV) assay (Campbell and Watson 2001). Briefly, after washing away alkaline phosphatase reaction reagents with double-distilled H_2_O, the plate was completely dried at RT. CV solution (0.1% in water, filtered) was added at 50 μL/well, incubated for 30 min at RT, and washed out with double-distilled H_2_O. Dye was released from the cells with 50 μL/well acetic acid (10% in water) at RT for 30 min. The A_590_ signal was then read in the microplate reader.

### Statistics.

Data were compared for significance of differences using Sigma Stat 3 (Jandel Scientific, San Rafael, CA) and one-way analysis of variance (significance accepted at *p* ≤0.05).

## Results

### Xenoestrogens can cause unique time-dependent patterns of ERK phosphorylation.

**Figure 1:**
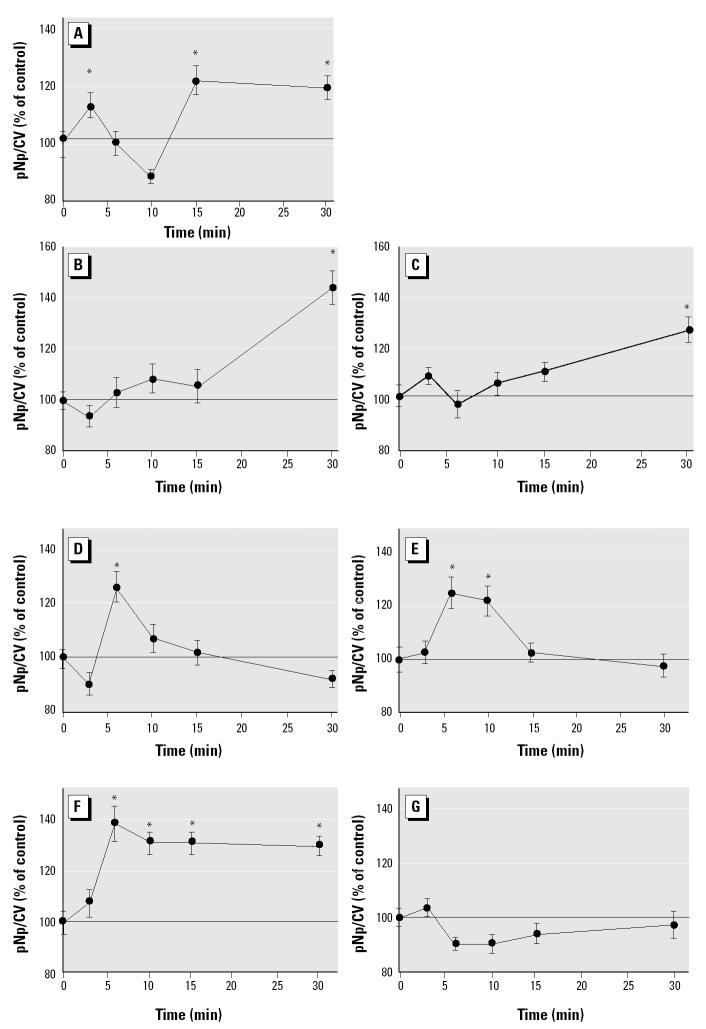
Time-dependent changes in the phosphorylation status of ERK. E_2_ (*A*), *p*-nonylphenol (*B*), endosulfan (*C*), DDE (*D*), dieldrin (*E*), coumestrol (*F*), and bisphenol A (*G*) were applied at 10^−9^ M. Data are presented as percentage of control values, which were set to 100; *n* = 48–60 wells/point taken from three different 96-well plates.
*Statistically significant (*p* < 0.05) compared with vehicle (0.0001% ethanol)-treated controls.

E_2_ (10^−9^ M) produced rapid (3, 15, and 30 min after application) and bimodal (with apparent periods of dephosphorylation between activation periods) ERK phosphorylation. Xenoestrogens at 10^−9^ M also caused ERK activations but with distinct temporal patterns (Figure 1). According to these patterns, compounds could be divided into several groups. Endosulfan and nonylphenol did not cause an initial (3 min) stimulation, but instead caused only a delayed single ERK phosphorylation peak at 30 min (which we designated slow-phase-only responders). DDE and dieldrin caused a single peak of activation at 6–10 min and were unable to cause a second sustained activation at 30 min (fast-phase-only responders). Coumestrol produced a rapid response (significant by 6 min), but the phosphorylation levels never declined after the activation, as was seen with the other active compounds. Bisphenol A did not produce any significant changes from the basal level of ERK phosphorylation during the 30 min assessment time and was not examined further in this study. All active xenoestrogens produced only a monophasic activation, failing to mimic the bimodal E_2_ activation.

### Xenoestrogens can be potent activators of ERK phosphorylation but with unique concentration-dependent patterns.

**Figure 2:**
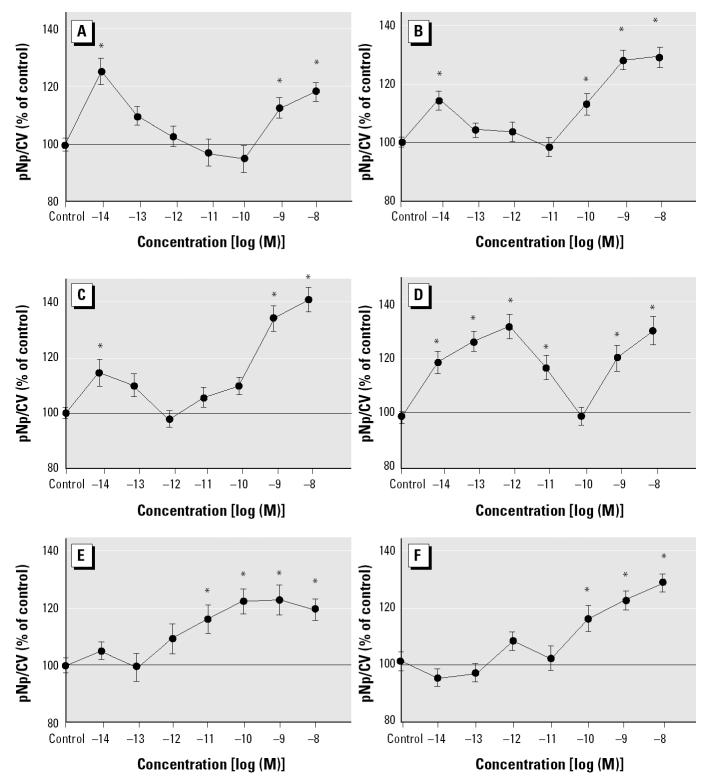
Concentration-dependent changes in the phosphorylation status of ERK. Each compound was tested at its previously determined time optimum (Figure 1): E_2_ (*A*) at 3 min, coumestrol (*B*) at 6 min, *p*-nonylphenol (*C*) and endosulfan (*D*) at 30 min, and DDE (*E*) and dieldrin (*F*) at 6 min. Data are presented as percentage (mean ± SE) of control values (which were set to 100); *n* = 78–85 wells from three different 96-well plates.
*Statistically significant (*p* < 0.05) compared with vehicle (0.0001% ethanol)-treated controls.

At optimal stimulation time points (Figure 1), different concentrations of E_2_ and xenoestrogens were compared in their ability to activate ERKs (Figure 2). E_2_ (tested at 3 min) was active in two concentration ranges: very low levels (10^−14^ M) and higher, but still physiological, levels (10^−9^–10^−8^ M). Nonylphenol and coumestrol showed similar patterns of potency, with dual ranges of activation similar to that seen with E_2_. Endosulfan was able to produce phosphorylation at almost all tested concentrations but still showed an apparent loss of activity centered on the 10^−10^ M concentration. DDE and dieldrin were not active at low concentrations (picomolar and lower) but were active in the concentration range centering on 10^−9^ M. Thus, although some subtle differences were observable between activation patterns for each compound, basically two patterns of stimulation were seen: compounds active in both the subpicomolar and nanomolar ranges (E_2_, endosulfan, nonylphenol, and coumestrol) versus compounds active only in the nanomolar range (DDE and dieldrin).

### Possible pathways for ERK activation for different compounds.

**Figure 3:**
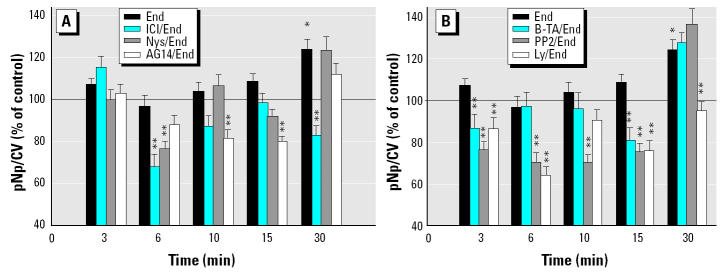
Effects of different inhibitors on endosulfan-induced ERK phosphorylation. (*A*) Inhibition effects for membrane-level components ICI, Nys, and AG 14. (*B*) Effects for postmembrane signaling system components B-TA, PP2, and Ly. Cells were pretreated with inhibitors at optimal effective concentrations and for optimal times of action: 1 μM ICI for 40 min, 50 μg/mL Nys for 40 min, 10 μM B-TA for 40 min, 10 μM PP2 for 20 min, 10 μM Ly for 40 min, 250 nM AG 14 for 20 min, or 0.01% DMSO vehicle (control) for 40 min, and then stimulated with endosulfan (End) at 1 nM, before the timed pERK plate assay. Values shown are mean ± SE; *n* = 40–90 wells from three to six different 96-well plates.
*Statistically significant (*p* < 0.05) compared with vehicle control. **Statistically significant (*p* < 0.05) compared with time-specific endosulfan-alone stimulated controls.

**Figure 4:**
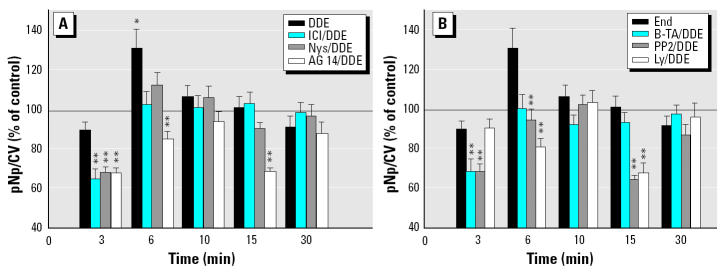
Effects of different inhibitors on DDE-induced ERK activation. (*A*) Inhibition effects for membrane-level components ICI, Nys, and AG 14. (*B*) Effects for postmembrane signaling system components B-TA, PP2, and Ly. Cells were pretreated with inhibitors at optimal effective concentrations and for optimal times of action: 1 μM ICI for 40 min, 50 μg/mL Nys for 40 min, 10 μM B-TA for 40 min, 10 μM PP2 for 20 min, 10 μM Ly for 40 min, 250 nM AG 14 for 20 min, or 0.01% DMSO vehicle (control) for 40 min, and then stimulated with DDE (1 nM). Values shown are mean ± SE; *n* = 45–85 wells from three to six different 96-well plates.
*Statistically significant (*p* < 0.05) compared with vehicle control. **Statistically significant (*p* < 0.05) compared with time-specific DDE-stimulated controls.

**Figure 5:**
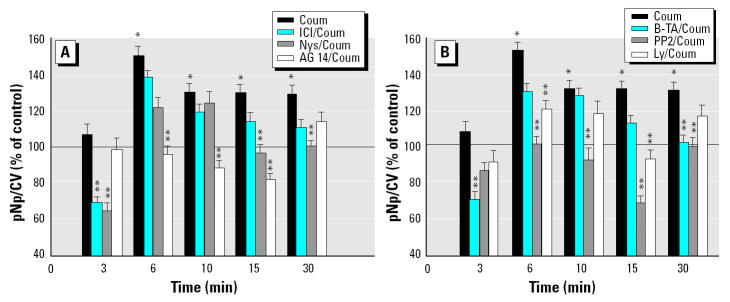
Effects of different inhibitors on coumestrol (Coum)-induced ERK activation. (*A*) Inhibition effects for membrane levels components ICI, Nys, and AG 14. (*B*) Effects for postmembrane signaling system components B-TA, PP2, and Ly. Cells were pretreated with inhibitors at optimal effective concentrations and for optimal times of action: 1 μM ICI for 40 min, 50 μg/mL Nys for 40 min, 10 μM B-TA for 40 min, 10 μM PP2 for 20 min, 10 μM Ly for 40 min, 250 nM AG 14 for 20 min, or 0.01% DMSO vehicle (control) for 40 min, and then stimulated with coumestrol at 1 nM. Values shown are mean ± SE; *n* = 39–79 wells from three to six 96-well plates.
*Statistically significant (*p* < 0.05) compared with vehicle control. **Statistically significant (*p* < 0.05) compared with time-specific coumestrol-stimulated values.

To detect possible signaling pathways through which E_2_ and xenoestrogens could affect pituitary tumor cells, we used inhibitors that have been described in the literature to pinpoint various mechanisms leading to ERK phosphorylation (Belcheva and Coscia 2002; Lowes et al. 2002). ICI and AG 14 are specific antagonists of estrogen and EGFRs, respectively. Nystatin (Nys) is a cholesterol-binding antibiotic that disrupts membrane architecture (Ushio-Fukai et al. 2001). B-TA is a Ca^2+^ chelator. PP2 is a Src kinase inhibitor, and Ly is a PI3K inhibitor. An example of each type of xenoestrogen based on temporal activation patterns shown in Figure 1 (fast-phase activator DDE, slow-phase activator endosulfan, and sustained-activator coumestrol) was examined for each of these inhibitor actions. All time points in their activation profiles were examined to determine when each mechanism might come into play (Figures 3–5). Inhibitor data were divided into two groups for clarity of presentation. Figures 3A, 4A, and 5A group together the responses to inhibitor compounds that can interfere with receptors (ERs, EGFRs) or disrupt membrane structures housing receptors: ICI, AG 14, and Nys (group A). Figures 3B, 4B, and 5B group together data for compounds whose substrates are mostly localized in the cell’s cytoplasm or are adjacent to the cell membrane and part of the downstream signaling cascades initiated at the membrane: B-TA, PP2, and Ly (group B).

Inhibition of endosulfan-stimulated ERK activation is shown in Figure 3. In these assays, endosulfan stimulated ERK significantly only at 30 min (as in Figure 1). Only ICI and Ly inhibited the endosulfan-provoked ERK activation at 30 min. The activity of ICI implicates ER-αin this process [because this subline does not express ER-β (Campbell and Watson 2001; Norfleet et al. 1999)]. However, even at times when endosulfan could not significantly elevate basal phosphorylation of ERK (3–15 min), all tested inhibitors were able to further deactivate basal ERK activity levels at some of these time points (e.g., AG 14 at 15 min; all group B compounds were effective at 3 and 15 min: PP2 at 6 and 10 min; Ly, ICI, and Nys at 6 min). Such inhibitions are xenoestrogen dependent because the inhibitors alone do not cause these dephosphorylations (Bulayeva et al. 2004).

DDE produced ERK activation only at 6 min (Figure 4), as expected from earlier studies (Figure 1). At this time point, ERK phosphorylation was inhibited by AG 14, PP2, and Ly. Although at other time points DDE did not raise ERK activation levels above basal, the addition of inhibitors nevertheless did lower activity to subbasal levels (all at 3 min; AG 14, PP2, and Ly at 15 min). Altogether, all tested compounds had an effect on basal ERK activity levels at some time point, but some tended to affect this outcome earlier in this time frame compared with others.

Coumestrol activated ERKs from 6 min onward in our assay (as shown in Figure 1F and in Figure 5). During the preactivation phase (3 min), basal levels of phosphorylation were further lowered by ICI, Nys, and B-TA. During the 6 min onward coumestrol activation phase, ICI was never effective at lowering ERK phosphorylation levels. AG 14 was effective at 6–15 min time points, and PP2 during the entire stimulation phase, which suggests early involvement of EGFR and Src kinase. Nys disruption of membrane structure (15–30 min) and Ly inhibition of PI3K (15 min) were effective only during these short temporal windows. B-TA’s chelation of Ca^2+^ was effective only very late in this sequence, at 30 min. Therefore, most inhibitors were effective at some point, although some later than others.

## Discussion

An important and surprising conclusion from our studies was that all tested estrogenic compounds, except bisphenol A, elicited rapid membrane-initiated actions at very low concentrations compared with their reported potencies in classical genomic pathways (Gutendorf and Westendorf 2001; Hodges et al. 2000; Inoue et al. 2002). All active compounds were able to produce rapid (3–30 min) ERK phosphorylations in the nanomolar concentration range, and some (E_2_, coumestrol, nonylphenol, and endosulfan) were also active in the subpicomolar range. Compounds from different classes of endocrine disruptors with dissimilar chemical structures (e.g., endosulfan as an organochlorine compound vs. nonylphenol as a simple phenolic detergent) can produce the same time-dependent activation pattern for ERKs. Coumestrol, a phytoestrogen, initiated a sustained ERK activation that had no temporal pattern similarity with any of the other tested compounds, including E_2_. None of the tested compounds was able to precisely repeat the E_2_ pattern of activation, which may contribute to their disruptive effects on estrogen-mediated endocrine functions.

The bimodal E_2_ time-dependent response seems to superimpose the patterns from both groups of other response-producing compounds: fast phase (during the first 10 min) and slow phase (not until 30 min). Interestingly, the most potent endocrine-disrupting chemical in genomic action assays, bisphenol A (Cappelletti et al. 2003; Recchia et al. 2004; Sato et al. 2003), was unable to produce time-dependent ERK activation. However, studies in progress show that bisphenol A, although unable to trigger ERK activation, nevertheless is somewhat effective at triggering Ca^2+^ influx, resulting in prolactin secretion (Wozniak et al., unpublished data). Thus, there are likely to be specific pathways within the nongenomic signaling network that individual compounds will trigger, leading to different functional end points. Therefore, each xenoestrogenic compound must be tested for an array of possible mechanistic routes of action.

Several tested xenoestrogenic compounds (coumestrol, nonylphenol, and endosulfan) demonstrated a bimodal dose–response curve for ERK activation similar to that seen with E_2_. This is reminiscent of the same bimodal dose–response pattern reported previously for rapid prolactin release after E_2_ (Watson et al. 1999b) and E_2_-BSA (Watson et al. 1995) treatment. The reason for this gap in dose responsiveness at intermediate concentrations is still not understood, but it is interesting that other estrogens in the present study demonstrate the same phenomenon. These very low effective doses for xenoestrogens demonstrate that many environmental contamination levels previously thought to be subtoxic may very well exert significant signal-and endocrine-disruptive effects, discernable only when the appropriate mechanism is assayed. Possible reasons for these potent effects not being noted previously are that little testing of the nongenomic pathway has been done, many tests did not examine such low concentrations, and some test conditions probably did not adequately remove endogenous estrogen levels (as we have done by use of low quantities of extensively charcoal-stripped serum) to reveal effects of these low concentrations. The potent effects we see on nongenomic signaling mechanisms could explain why concentrations previously determined to be inactive via genomic mechanisms still have toxic and teratogenic effects on wildlife (Brucker-Davis et al. 2001). Therefore, the threat levels of these compounds to wildlife, and probably humans, need to be reconsidered.

The complexity of multiple signaling pathways triggered simultaneously is probably related to the organization of ERs within membrane substructures (caveolae or membrane rafts), where they encounter many signaling machineries (Chambliss et al. 2000; Nadal et al. 2000; Razandi et al. 2002). Our data indicate that the disruption of a nongenomic signaling cascade midway in its time course caused by Nys (e.g., for coumestrol) probably corresponds to disruption of this cholesterol-rich meeting place for ligands and receptors with their downstream signaling partners. Interestingly, only endosulfan effects failed to be inhibited by disruption of cholesterol-rich membrane structure, perhaps implicating different membrane subdomains as locations for the actions of different compounds. Alternatively, endosulfan signaling may move into the intracellular compartment rapidly after initiation and earlier than 3 min (and earlier time point assessment using these methods would be technically difficult).

Although here we have only directly assessed ERK activation as a signaling cascade end point, the participation of upstream signaling repertoires was implicated by our specific inhibitor assays. We found that all examined pathways can participate in ERK activation but that different xenoestrogens use different subsets of these pathways. Table 1 summarizes the vulnerability of E_2_- (Bulayeva et al. 2004), endosulfan-, DDE- and coumestrol-initiated actions to inhibitors of different signaling components. E_2_- or xenoestrogen-treated cells showed inhibitions of both stimulated ERK phosphorylation levels and background levels of phosphorylation. Our time course measurements allowed an analysis of when pathway inhibitions affected the outcome of ERK phosphorylation, and we noted whether this was very early after treatment (3 min) or later (≥6 min). Although these times are arbitrary cutoffs, they allowed us to highlight some possible temporal differences in the effects of compounds’ pathways. All xenoestrogens shared activation via all pathways, although compounds differed in their timing of pathway engagement. For example, inhibitors of action via the EGFR and ER were sometimes effective only after 6 min. This could mean that the activation sequence took some time to reach the level of a receptor (EGFR is downstream) or that a unique conformation of receptors in the plasma membrane could initially prevent binding by antagonists (ER). Although all xenoestrogens shared activation via the PI3K pathway, PI3K inhibitors could not lower DDE- or coumesterol-mediated ERK phosphorylation levels until ≥6 min, so perhaps progression to this level of signaling took variable amounts of time depending upon the compound initiating the response. A possible complication to our interpretation of these data is the recent demonstration that Ly can have antiestrogenic activity by binding to ER Pasapera Limon et al. 2003).

Inhibitors also interfered with ERK phosphorylation levels that were not stimulated by xenoestrogens above untreated background levels. For example, endosulfan, which elevates ERK phosphorylation only after 30 min, still participated in a significant lowering of basal ERK phosphorylation levels at early time points. In our previous work (Bulayeva et al. 2004), we demonstrated that these inhibitors by themselves were unable to change basal levels of ERK phosphorylation; the present study thus shows that the presence of xenoestrogens was necessary to produce inhibitor-driven decreases below basal levels. Because ERK phosphorylation demonstrated a complex temporal fluctuation, we speculate that periods of “dephosphorylation” demonstrated by our data could be the result of desensitization of the stimulatory pathways and/or phosphatase activation. Such deactivation and reactivation profiles may be very important for specific estrogenic stimulatory effects because other hormones and regulators are known to operate in an oscillatory fashion through kinase inactivation (MacDonald et al. 1997) or protein degradation (Murray 2004). However, the rapid recovery times in our pattern argue against the latter mechanism.

Wide diversity in signaling cascades leading to ERK activation can perhaps be explained by the nature of mERs and the probable necessity of their interactions with many other different signaling partners. Xenoestrogens are highly diverse in structure, and the conformation of different xenoestrogen–ER complexes could be significantly different from that of an E_2_–ER complex (Brzozowski et al. 1997; Suzuki et al. 2001; van Lipzig et al. 2004). This could alter the receptor protein’s surface topography and thus its interactions with partner proteins, as has been demonstrated for ligand effects on nuclear receptor interactions with coactivators and corepressors. The nature and magnitude of responses are probably a function of the conformation the receptor assumes around these diverse molecules and the repertoire of interacting proteins present in different cell types (so pituitary cell patterns may not be predictive for other cell types). The outcomes can be different and multiplex. Therefore, xenoestrogens will need to be individually examined for these complex mechanistic and functional outcomes in different tissues.

## Figures and Tables

**Table 1:** Xenoestrogens each have unique signaling pathway inhibition patterns during rapidly initiated estrogenic actions.

Inhibitors	E_2_	Endosulfan	DDE	Coumestrol
Ca^2+^	+	+	+	+
Membrane	+		+	+
Src	+	+	+	(+)
PI3K	+	+	(+)	(+)
EGFR	(+)	(+)	+	(+)
ER	(+)	(+)	+	+

+, inhibition effect at 3 min; (+), inhibition effect at ≥6 min. For comparison, the E_2_ response inhibition data summarized here are taken from Bulayeva et al. (2004). All time points where the combination of xenoestrogen and inhibitor showed a significant reduction in ERK phosphorylation levels below the hormone-untreated background level are shown.

## References

[b1-ehp0112-001481] Adeoya-Osiguwa SA, Markoulaki S, Pocock V, Milligan SR, Fraser LR (2003). 17beta-Estradiol and environmental estrogens significantly affect mammalian sperm function. Hum Reprod.

[b2-ehp0112-001481] Aronica SM, Kraus WL, Katzenellenbogen BS (1994). Estrogen action via the cAMP signaling pathway: stimulation of adenylate cyclase and cAMP-regulated gene transcription. Proc Natl Acad Sci USA.

[b3-ehp0112-001481] Belcheva MM, Coscia CJ (2002). Diversity of G protein-coupled receptor signaling pathways to ERK/MAP kinase. Neurosignals.

[b4-ehp0112-001481] Bonefeld-Jorgensen EC, Andersen HR, Rasmussen TH, Vinggaard AM (2001). Effect of highly bioaccumulated poly-chlorinated biphenyl congeners on estrogen and androgen receptor activity. Toxicology.

[b5-ehp0112-001481] Brucker-Davis F, Thayer K, Colborn T (2001). Significant effects of mild endogenous hormonal changes in humans: considerations for low-dose testing. Environ Health Perspect.

[b6-ehp0112-001481] Brzozowski AM, Pike AC, Dauter Z, Hubbard RE, Bonn T, Engstrom O (1997). Molecular basis of agonism and antagonism in the oestrogen receptor. Nature.

[b7-ehp0112-001481] Bulayeva NN, Gametchu B, Watson CS (2004). Quantitative measurement of estrogen-induced ERK 1 and 2 activation via multiple membrane-initiated signaling pathways. Steroids.

[b8-ehp0112-001481] Campbell CH, Watson CS (2001). A comparison of membrane vs. intracellular estrogen receptor-αin GH_3_/B6 pituitary tumor cells using a quantitative plate immunoassay. Steroids.

[b9-ehp0112-001481] Cappelletti V, Saturno G, Miodini P, Korner W, Daidone MG (2003). Selective modulation of ER-beta by estradiol and xenoestrogens in human breast cancer cell lines. Cell Mol Life Sci.

[b10-ehp0112-001481] Chambliss KL, Yuhanna IS, Mineo C, Liu P, German Z, Sherman TS (2000). Estrogen receptor alpha and endothelial nitric oxide synthase are organized into a functional signaling module in caveolae. Circ Res.

[b11-ehp0112-001481] Chen Z, Yuhanna IS, Galcheva-Gargova Z, Karas RH, Mendelsohn RE, Shaul PW (1999). Estrogen receptor alpha mediates the nongenomic activation of endothelial nitric oxide synthase by estrogen. J Clin Invest.

[b12-ehp0112-001481] Cossette LJ, Gaumond I, Martinoli MG (2002). Combined effect of xenoestrogens and growth factors in two estrogen-responsive cell lines. Endocrine.

[b13-ehp0112-001481] Doolan CM, Harvey BJ (2003). A Gαs protein-coupled membrane receptor, distinct from the classical oestrogen receptor, transduces rapid effects of oestradiol on [Ca^2+^]_i_ in female rat distal colon. Mol Cell Endocrinol.

[b14-ehp0112-001481] Elsby R, Maggs JL, Ashby J, Paton D, Sumpter JP, Park BK (2001). Assessment of the effects of metabolism on the estrogenic activity of xenoestrogens: a two-stage approach coupling human liver microsomes and a yeast estrogenicity assay. J Pharmacol Exp Ther.

[b15-ehp0112-001481] English J, Pearson G, Wilsbacher J, Swantek J, Karandikar M, Xu S (1999). New insights into the control of MAP kinase pathways. Exp Cell Res.

[b16-ehp0112-001481] Filardo EJ, Quinn JA, Bland KI, Frackelton AR (2000). Estrogen-induced activation of Erk-1 and Erk-2 requires the G protein-coupled receptor homolog, GPR30, and occurs via trans-activation of the epidermal growth factor receptor through release of HB-EGF. Mol Endocrinol.

[b17-ehp0112-001481] Granek V, Rishpon J (2002). Detecting endocrine-disrupting compounds by fast impedance measurements. Environ Sci Technol.

[b18-ehp0112-001481] Gutendorf B, Westendorf J (2001). Comparison of an array of in vitro assays for the assessment of the estrogenic potential of natural and synthetic estrogens, phytoestrogens and xenoestrogens. Toxicology.

[b19-ehp0112-001481] Haynes MP, Li L, Sinha D, Russell KS, Hisamoto K, Baron R (2003). Src kinase mediates phosphatidylinositol 3-kinase/Akt-dependent rapid endothelial nitric-oxide synthase activation by estrogen. J Biol Chem.

[b20-ehp0112-001481] Hodges LC, Bergerson JS, Hunter DS, Walker CL (2000). Estrogenic effects of organochlorine pesticides on uterine leiomyoma cells in vitro. Toxicol Sci.

[b21-ehp0112-001481] Inoue A, Hayashi S, Aoyagi K, Nishigaki M, Sasaki H, Kiyama R (2002). A reporter gene assay for evaluation of tissue-specific responses to estrogens based on the differential use of promoters A to F of the human estrogen receptor alpha gene. J Pharmacol Toxicol Methods.

[b22-ehp0112-001481] Kelly MJ, Lagrange AH, Wagner EJ, Ronnekleiv OK (1999). Rapid effects of estrogen to modulate G protein-coupled receptors via activation of protein kinase A and protein kinase C pathways. Steroids.

[b23-ehp0112-001481] Krishnan AV, Stathis P, Permuth SF, Tokes L, Feldman D (1993). Bisphenol-A: an estrogenic substance is released from polycarbonate flasks during autoclaving. Endocrinology.

[b24-ehp0112-001481] Lee HS, Miyauchi K, Nagata Y, Fukuda R, Sasagawa S, Endoh H (2002). Employment of the human estrogen receptor beta ligand-binding domain and co-activator SRC1 nuclear receptor-binding domain for the construction of a yeast two-hybrid detection system for endocrine disrupters. J Biochem (Tokyo).

[b25-ehp0112-001481] Levin ER (1999). Cellular functions of the plasma membrane estrogen receptor. Trends Endocrinol Metab.

[b26-ehp0112-001481] Long XH, Steinmetz R, Ben Jonathan N, Caperell-Grant A, Young PCM, Nephew KP (2000). Strain differences in vaginal responses to the xenoestrogen bisphenol A. Environ Health Perspect.

[b27-ehp0112-001481] Lowes VL, Ip NY, Wong YH (2002). Integration of signals from receptor tyrosine kinases and g protein-coupled receptors. Neurosignals.

[b28-ehp0112-001481] MacDonald MJ, Al Masri H, Jumelle-Laclau M, Cruz MO (1997). Oscillations in activities of enzymes in pancreatic islet subcellular fractions induced by physiological concentrations of effectors. Diabetes.

[b29-ehp0112-001481] Massaad C, Barouki R (1999). An assay for the detection of xenoestrogens based on a promoter containing overlapping EREs. Environ Health Perspect.

[b30-ehp0112-001481] McLachlan JA (1993). Functional toxicology: a new approach to detect biologically active xenobiotics. Environ Health Perspect.

[b31-ehp0112-001481] Murray AW (2004). Recycling the cell cycle: cyclins revisited. Cell.

[b32-ehp0112-001481] Nadal A, Ropero AB, Laribi O, Maillet M, Fuentes E, Soria B (2000). Nongenomic actions of estrogens and xenoestrogens by binding at a plasma membrane receptor unrelated to estrogen receptor alpha and estrogen receptor beta. Proc Natl Acad Sci USA.

[b33-ehp0112-001481] Nadal A, Rovira JM, Laribi O, Leonquinto T, Andreu E, Ripoll C (1998). Rapid insulinotropic effect of 17-β-estradiol via a plasma membrane receptor. FASEB J.

[b34-ehp0112-001481] Norfleet AM, Thomas ML, Gametchu B, Watson CS (1999). Estrogen receptor-αdetected on the plasma membrane of aldehyde-fixed GH_3_/B6/F10 rat pituitary cells by enzyme-linked immunocytochemistry. Endocrinology.

[b35-ehp0112-001481] Pappas TC, Gametchu B, Yannariello-Brown J, Collins TJ, Watson CS (1994). Membrane estrogen receptors in GH_3_/B6 cells are associated with rapid estrogen-induced release of prolactin. Endocrine.

[b36-ehp0112-001481] Pasapera Limon AM, Herrera-Munoz J, Gutierrez-Sagal R, Ulloa-Aguirre A (2003). The phosphatidylinositol 3-kinase inhibitor LY294002 binds the estrogen receptor and inhibits 17beta-estradiol-induced transcriptional activity of an estrogen sensitive reporter gene. Mol Cell Endocrinol.

[b37-ehp0112-001481] Prevot V, Croix D, Rialas CM, Poulain P, Fricchione GL, Stefano GB (1999). Estradiol coupling to endothelial nitric oxide stimulates gonadotropin-releasing hormone release from rat median eminence via a membrane receptor. Endocrinology.

[b38-ehp0112-001481] Rajapakse N, Silva E, Kortenkamp A (2002). Combining xenoestrogens at levels below individual no-observed-effect concentrations dramatically enhances steroid hormone action. Environ Health Perspect.

[b39-ehp0112-001481] Razandi M, Oh P, Pedram A, Schnitzer J, Levin ER (2002). ERs associate with and regulate the production of caveolin: implications for signaling and cellular actions. Mol Endocrinol.

[b40-ehp0112-001481] Razandi M, Pedram A, Park ST, Levin ER (2003). Proximal events in signaling by plasma membrane estrogen receptors. J Biol Chem.

[b41-ehp0112-001481] Recchia AG, Vivacqua A, Gabriele S, Carpino A, Fasanella G, Rago V (2004). Xenoestrogens and the induction of proliferative effects in breast cancer cells via direct activation of oestrogen receptor alpha. Food Addit Contam.

[b42-ehp0112-001481] Rosselli M, Reinhart K, Imthurn B, Keller PJ, Dubey RK (2000). Cellular and biochemical mechanisms by which environmental oestrogens influence reproductive function. Hum Reprod Update.

[b43-ehp0112-001481] Rousseau J, Cossette L, Grenier S, Martinoli MG (2002). Modulation of prolactin expression by xenoestrogens. Gen Comp Endocrinol.

[b44-ehp0112-001481] Ruehlmann DO, Steinert JR, Valverde MA, Jacob R, Mann GE (1998). Environmental estrogenic pollutants induce acute vascular relaxation by inhibiting L-type Ca2+ channels in smooth muscle cells. FASEB J.

[b45-ehp0112-001481] Sato K, Matsuki N, Ohno Y, Nakazawa K (2003). Estrogens inhibit l-glutamate uptake activity of astrocytes via membrane estrogen receptor alpha. J Neurochem.

[b46-ehp0112-001481] Sonnenschein C, Soto AM, Fernandez MF, Olea N, Olea-Serrano MF, Ruiz-Lopez MD (1995). Development of a marker of estrogenic exposure in human serum. Clin Chem.

[b47-ehp0112-001481] Soto AM, Chung KL, Sonnenschein C (1994). The pesticides endosulfan, toxaphene, and dieldrin have estrogenic effects on human estrogen-sensitive cells. Environ Health Perspect.

[b48-ehp0112-001481] Suzuki T, Ide K, Ishida M, Shapiro S (2001). Classification of environmental estrogens by physicochemical properties using principal component analysis and hierarchical cluster analysis. J Chem Inf Comput Sci.

[b49-ehp0112-001481] Tsai KS, Yang RS, Liu SH (2004). Benzo[a]pyrene regulates osteoblast proliferation through an estrogen receptor-related cyclooxygenase-2 pathway. Chem Res Toxicol.

[b50-ehp0112-001481] Turner KO, Syvanen M, Meizel S (1997). The human acrosome reaction is highly sensitive to inhibition by cyclodiene insecticides. J Androl.

[b51-ehp0112-001481] Ushio-Fukai M, Hilenski L, Santanam N, Becker PL, Ma Y, Griendling KK (2001). Cholesterol depletion inhibits epidermal growth factor receptor transactivation by angiotensin II in vascular smooth muscle cells: role of cholesterol-rich microdomains and focal adhesions in angiotensin II signaling. J Biol Chem.

[b52-ehp0112-001481] van Lipzig MM, ter Laak AM, Jongejan A, Vermeulen NP, Wamelink M, Geerke D (2004). Prediction of ligand binding affinity and orientation of xenoestrogens to the estrogen receptor by molecular dynamics simulations and the linear interaction energy method. J Med Chem.

[b53-ehp0112-001481] Watson CS, Campbell CH, Gametchu B (1999a). Membrane estrogen receptors on rat pituitary tumor cells: immuno-identification and responses to estradiol and xenoestrogens. Exp Physiol.

[b54-ehp0112-001481] Watson CS, Norfleet AM, Pappas TC, Gametchu B (1999b). Rapid actions of estrogens in GH_3_/B6 pituitiary tumor cells via a plasma membrane version of estrogen receptor-γ. Steroids.

[b55-ehp0112-001481] Watson CS, Pappas TC, Gametchu B (1995). The other estrogen receptor in the plasma membrane: implications for the actions of environmental estrogens. Environ Health Perspect.

[b56-ehp0112-001481] Willard SP, Frawley LS (1998). Phytoestrogens have agonistic and combinatorial effects on estrogen-responsive gene expression in MCF-7 human breast cancer cells. Endocrine.

[b57-ehp0112-001481] Witorsch RJ (2002). Endocrine disruptors: can biological effects and environmental risks be predicted?. Regul Toxicol Pharmacol.

[b58-ehp0112-001481] Wober J, Weisswange I, Vollmer G (2002). Stimulation of alkaline phosphatase activity in Ishikawa cells induced by various phytoestrogens and synthetic estrogens. J Steroid Biochem Mol Biol.

[b59-ehp0112-001481] Yu SJ, Keenan SM, Tong W, Welsh WJ (2002). Influence of the structural diversity of data sets on the statistical quality of three-dimensional quantitative structure-activity relationship (3D-QSAR) models: predicting the estrogenic activity of xenoestrogens. Chem Res Toxicol.

